# CNS-targeted glucocorticoid reduces pathology in mouse model of amyotrophic lateral sclerosis

**DOI:** 10.1186/2051-5960-2-66

**Published:** 2014-06-13

**Authors:** Matthew C Evans, Pieter J Gaillard, Marco de Boer, Chantal Appeldoorn, Rick Dorland, Nicola R Sibson, Martin R Turner, Daniel C Anthony, Helen B Stolp

**Affiliations:** Nuffield Department of Clinical Neurosciences, University of Oxford, Oxford, OX3 9DU UK; to-BBB technologies BV, Leiden, the Netherlands; CR-UK/MRC Gray Institute for Radiation, Oncology and Biology, Department of Oncology, University of Oxford, Oxford, OX3 7LJ UK; Department of Pharmacology, University of Oxford, Mansfield Road, Oxford, OX1 3QT UK; Department of Physiology, Anatomy and Genetics, University of Oxford, Oxford, OX1 3QX UK

**Keywords:** *SOD1*^*G93A*^, Steroids, MRI, Astrocytes, Vacuolation

## Abstract

**Background:**

Hallmarks of CNS inflammation, including microglial and astrocyte activation, are prominent features in post-mortem tissue from amyotrophic lateral sclerosis (ALS) patients and in mice overexpressing mutant *superoxide dismutase-1* (*SOD1*^*G93A*^). Administration of non-targeted glucocorticoids does not significantly alter disease progression, but this may reflect poor CNS delivery. Here, we sought to discover whether CNS-targeted, liposomal encapsulated glucocorticoid would inhibit the CNS inflammatory response and reduce motor neuron loss. *SOD1*^*G93A*^ mice were treated with saline, free methylprednisolone (MP, 10 mg/kg/week) or glutathione PEGylated liposomal MP (2B3-201, 10 mg/kg/week) and compared to saline treated wild-type animals. Animals were treated weekly with intravenous injections for 9 weeks from 60 days of age. Weights and motor performance were monitored during this period. At the end of the experimental period (116 days) mice were imaged using *T*_2_-weighted MRI for brainstem pathology; brain and spinal cord tissue were then collected for histological analysis.

**Results:**

All *SOD1*^*G93A*^ groups showed a significant decrease in motor performance, compared to baseline, from ~100 days. *SOD1*^*G93A*^ animals showed a significant increase in signal intensity on *T*_2_ weighted MR images, which may reflect the combination of neuronal vacuolation and glial activation in these motor nuclei. Treatment with 2B3-201, but not free MP, significantly reduced *T*_2_ hyperintensity observed in *SOD1*^*G93A*^ mice. Compared to saline-treated and free-MP-treated *SOD1*^*G93A*^ mice, those animals given 2B3-201 displayed significantly improved histopathological outcomes in brainstem motor nuclei, which included reduced gliosis and neuronal loss.

**Conclusions:**

In contrast to previous reports that employed free steroid preparations, CNS-targeted anti-inflammatory agent 2B3-201 (liposomal methylprednisolone) has therapeutic potential, reducing brainstem pathology in the *SOD1*^*G93A*^ mouse model of ALS. 2B3-201 reduced neuronal loss and vacuolation in brainstem nuclei, and reduced activation preferentially in astrocytes compared with microglia. These data also suggest that other previously ineffective therapies could be of therapeutic value if delivered specifically to the CNS.

## Background

Amyotrophic lateral sclerosis (ALS) is the most prevalent type of motor neuron disease [1]; the *SOD1*^*G93A*^ mouse [2] closely models the progressive motor neuron pathology of human ALS and has been useful for the study of mechanisms of selective motor neuron death. Neuronal degeneration and tissue vacuolation is accompanied by changes in magnetic resonance imaging (MRI) parameters [3, 4] leading to the possibility of using MRI as an *in vivo* biomarker of pathology in addition to conventional behavioural and histological endpoints. In particular we have shown previously that T_2_ MRI is a sensitive pathological biomarker in *SOD1*^*G93A*^ mice [5].

The *SOD1*^*G93A*^ mouse displays astrocyte and microglial activation, which are established hallmarks of a CNS inflammatory response [6], and are well-recognised in individuals with ALS [7–9]. Astrocytes and microglia become activated pre-symptomatically, and this becomes more widespread and evident as the disease progresses [10, 11]. The inflammatory response in ALS, which correlates with disease progression [12], has provoked the trial of a number of anti-inflammatory agents as potential disease-modifying agents [13–16]. However, none of these therapeutic approaches have translated to the clinic. In particular, despite preclinical studies which suggest that steroids might be of value in the treatment of ALS [17], a study investigating the therapeutic potential of prednisolone and azathioprine in a mixed population of motor neuron disease patients, including ALS patients, found only limited differences between treatment groups [18]. It therefore remains contentious whether corticosteroids could afford some therapeutic benefit in ALS. Improved delivery of anti-inflammatory agents to the CNS may allow more definitive assessment of the therapeutic potential of these drugs in ALS.

The blood–brain barrier (BBB) regulates the entrance of many lipophilic substances, including steroids into the CNS. Studies indicate that BBB penetrance for methylprednisolone is lower than expected, due to efflux from the CNS by p-glycoprotein [19]. Therefore, increasing the sustained delivery of steroid compounds into the CNS may be a way to maximize the therapeutic effect, while reducing side effects from the increased blood concentrations required to achieve adequate CNS delivery. Glutathione PEGylated liposomal methylprednisolone (2B3-201) overcomes these problems by changing the pharmacokinetics and the biodistribution of the drug and, most importantly, targeting endogenous glutathione transporters at the BBB [20]. This drug delivery system has been used successfully in a murine model of multiple sclerosis [21], where it was found that 2B3-201 reduced clinical severity significantly more than either non-targeted liposomes or unpackaged methylprednisolone, and empty targeted liposomes had no effect on the disease severity. Therefore this effect can be attributed to the sustained enhanced delivery of methylprednisolone into the CNS. Thus we sought to discover whether 2B3-201 would be more effective than free steroid at improving the MRI, behavioural and histological endpoints in the *SOD1*^*G93A*^ model of ALS.

## Results

### Behaviour and weights

**Figure 1:**
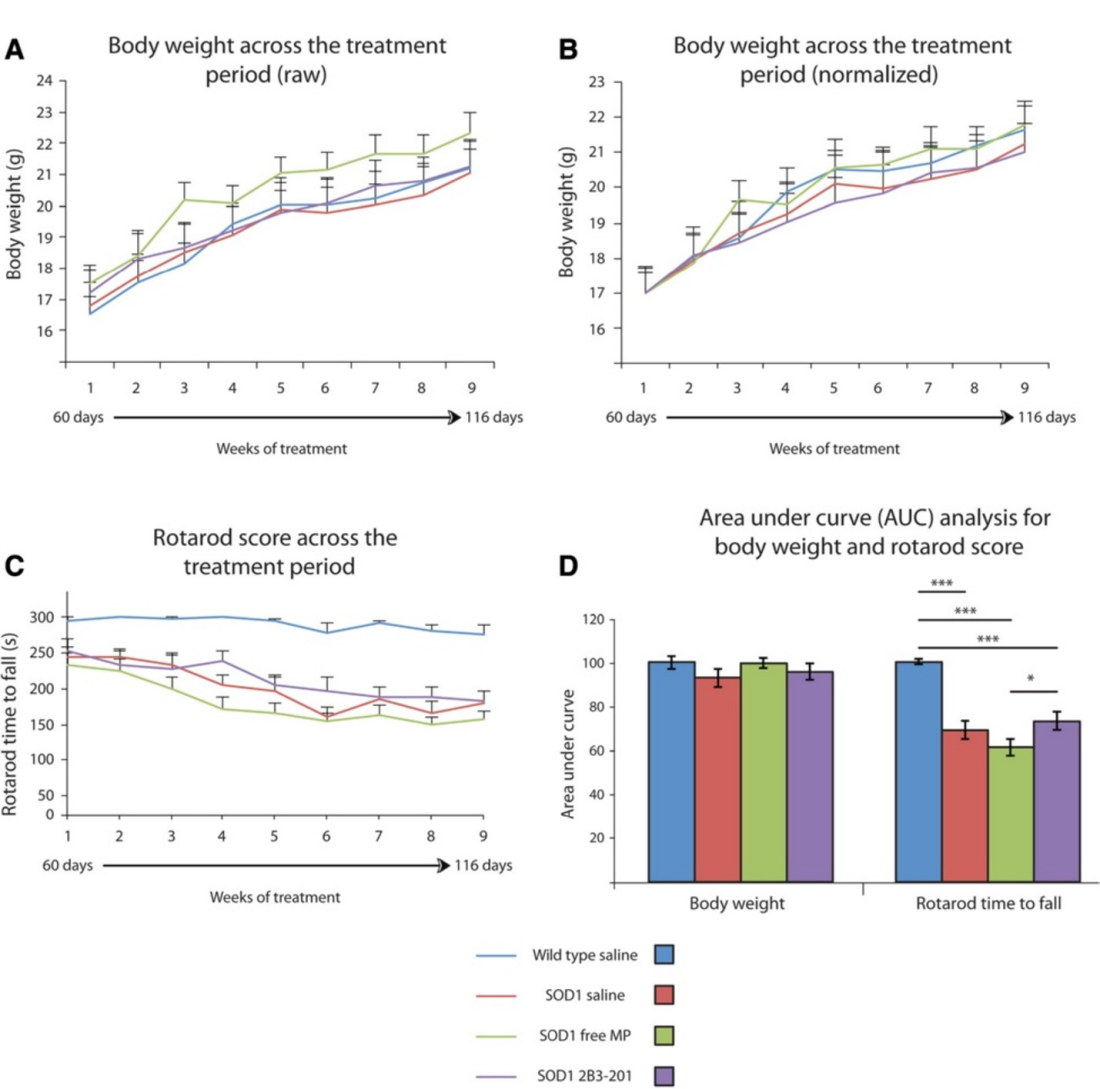
**Disease progression assessed by weight and motor behaviour.** Weight data (unadjusted – **A**; normalized at 60 days – **B)** shows a gradual increase in weight in all groups, with no significant difference between genotypes or treatment groups at any time point. Rotarod scores **(C)** were higher at all time points for WT compared with all *SOD1* ^*G93A*^ treatment groups, and all *SOD1* ^*G93A*^ groups showed a typical decline over time, with no significant difference between any of the treatment groups. There was no significant difference between groups for weight data with area-under-the-curve analysis **(D)**; *SOD1* ^*G93A*^ 2B3-201 had significantly greater area under the curve (increased motor scores) compared with free MP, but there was no difference when compared with *SOD1* ^*G93A*^ saline. Data are presented as mean ± standard error. *** p<0.001.

The mean weight of all groups of mice increased over time, and no significant difference between any of the groups was noted at any time point (Figure 1 – A, B) (ANOVA p>0.05). Owing to the variation in weights at baseline between the groups (Figure 1A), we performed the analysis after accounting for baseline weight (Figure 1B). However, there was still no significant difference between groups after this normalization (ANOVA p>0.05). We confirmed this for the whole time course using area under the curve (AUC) analysis (Figure 1 – D, left) (p>0.05).

For the rotarod test, a significant effect of time-point was found (p < 0.001) together with a significant interaction between time and treatment (p < 0.01). WT mice remained on the apparatus for longer than all of the *SOD1*^*G93A*^ groups at all time points (Figure 1 – C) (p < 0.05). A decline in motor performance was evident as the disease progressed in all *SOD1*^*G93A*^ mice, and reached significance from baseline (p < 0.05) between 80 and 95 days (*SOD1*^*G93A*^ saline, 95 days; *SOD1*^*G93A*^ free MP, 81 days; *SOD1*^*G93A*^ 2B3-201, 88 days). No difference was found between *SOD1*^*G93A*^ treatment groups at any time point (p > 0.05). AUC analysis (Figure 1 – D, right) also showed a significant difference between groups (ANOVA p < 0.001). Post-hoc comparisons revealed a significant difference between WT and all *SOD1*^*G93A*^ treatment groups (p < 0.001). In *SOD1*^*G93A*^ mice treated with 2B3-201, a significantly greater AUC was found compared with *SOD1*^*G93A*^ mice treated with free MP (p<0.05), but no significant difference was seen between any of the other *SOD1*^*G93A*^ treatment comparisons (p>0.05).

### T_2_ MRI and tissue vacuolation

**Figure 2:**
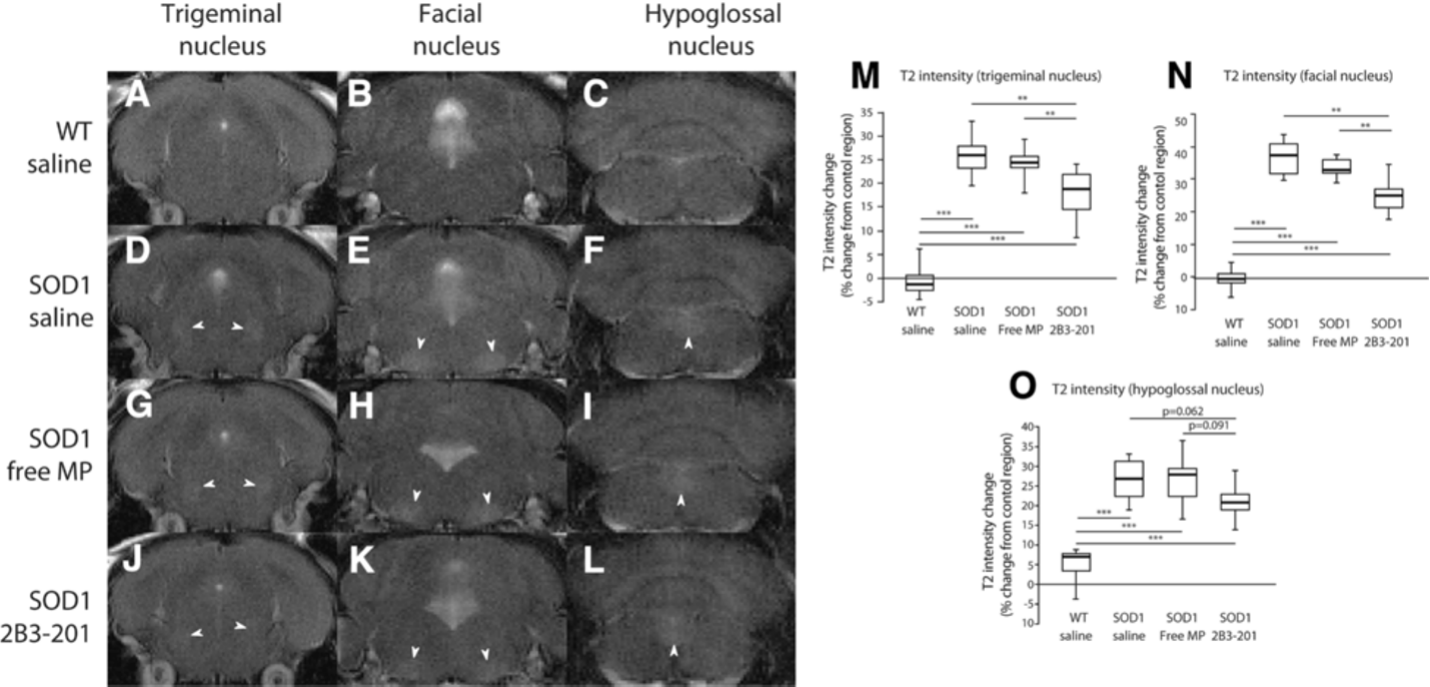
**Changes in** ***T***_**2**_ **MR signal intensity in cranial motor nuclei following steroid treatment.** An increase in signal is present in the trigeminal (V), facial (VII) and hypoglossal (XII) nuclei in *SOD1* ^*G93A*^ saline mice **(D-F)** compared to WT animals **(A-C)**. A comparable signal increase in seen for *SOD1* ^*G93A*^ mice treated with free MP **(G-I)**, but in comparison to saline and free MP treated *SOD1* ^*G93A*^ mice, those treated with 2B3-201 **(J-L)** show a markedly reduced signal intensity in these three motor nuclei. These data are presented graphically for the trigeminal, facial and hypoglossal nuclei in **M-O** respectively. * p < 0.05; ** p<0.01; *** p<0.001.

To discover whether 2B3-201 treatment had any effect on brainstem pathology *in vivo*, mice were scanned with *T*_2_ MRI. *SOD1*^*G93A*^ mice treated with saline had increased *T*_2_ signal intensity in the facial nucleus compared to surrounding grey matter (median 37.3 [interquartile range 9.2]%) (Figure 2 – B, E, H, K, N), which was significantly greater than *T*_2_ intensity in WT mice (p < 0.001), where no such signal change was seen. *SOD1*^*G93A*^ mice treated with free MP showed a similar increase in signal intensity (32.5 [3.9]%), which was not significantly different from *SOD1*^*G93A*^ treated with saline (p > 0.05). However, the signal increase in *SOD1*^*G93A*^ mice treated with 2B3-201 was attenuated (24.8 [5.8]%), and was significantly lower than both *SOD1*^*G93A*^ mice treated with saline and mice treated with free MP (p < 0.01). The same pattern was present in both the trigeminal (Figure 2 – A, D, G, J, M) and hypoglossal nuclei (Figure 2 – C, F, I, L, O), with attenuation in signal intensity for *SOD1*^*G93A*^ mice treated with 2B3-201 compared with saline and free MP.

### Histological confirmation of vacuolation

**Figure 3:**
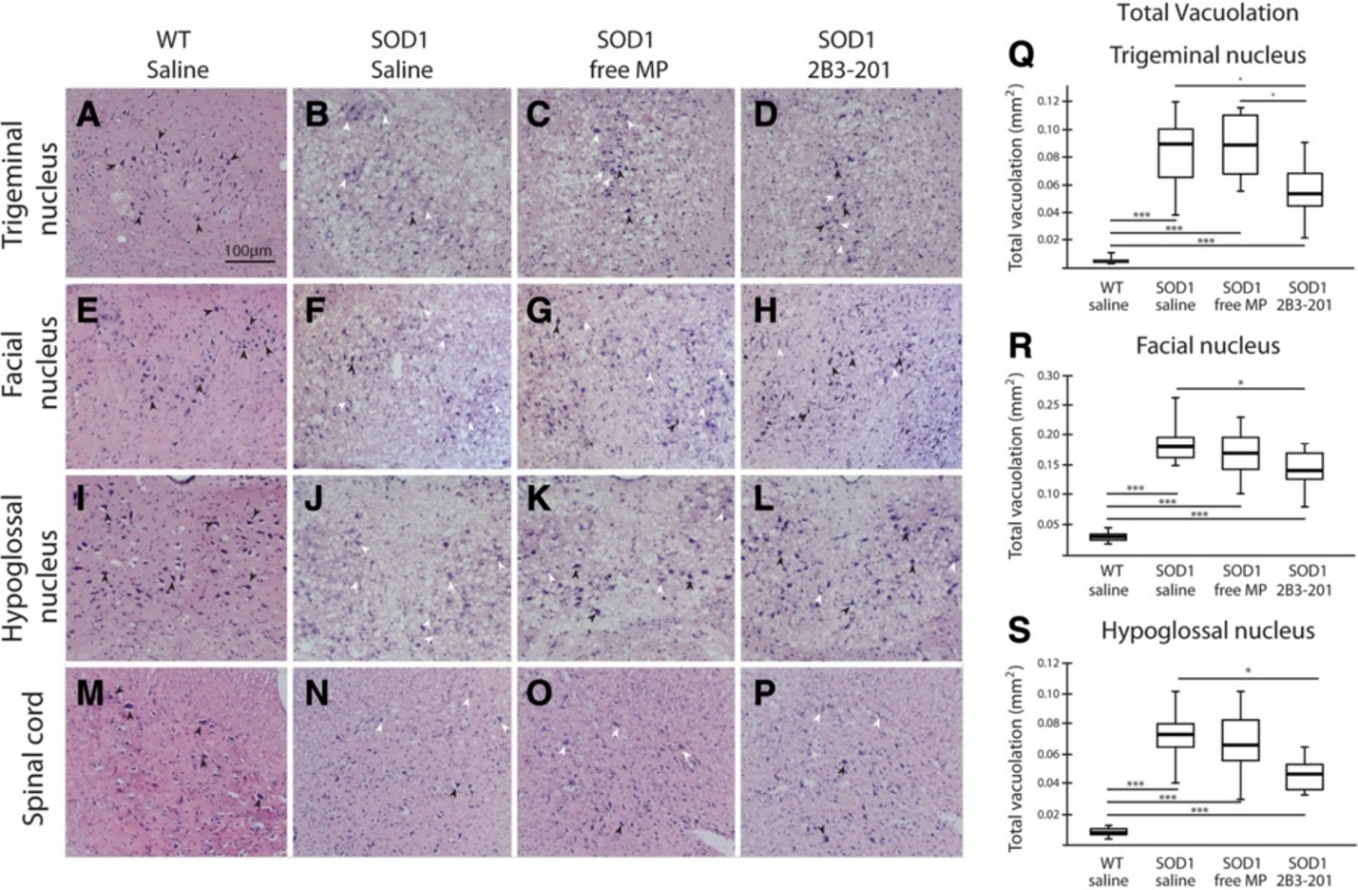
**Mutant** ***SOD1*** **-induced vacuolation is ameliorated by steroid treatment.** WT tissue shows staining of large motor neurons (black arrows) in the cranial motor nuclei **(A, E, I)** and spinal cord **(M)**, with no structural abnormalities. In contrast, *SOD1* ^*G93A*^ saline **(B, F, J, N)** and *SOD1* ^*G93A*^ free MP **(C, G, K, O)** show a reduction in large motor neurons, with more morphologically abnormal neurons (white arrows). There is also substantial vacuolation. *SOD1* ^*G93A*^ mice treated with 2B3-201 **(D, H, L, P)** have reduced vacuolation in the cranial motor nuclei **(Q-S)**. Scale bar applies to all images. Images shown are representative median values in the graphs. * p<0.05; ** p<0.01; *** p<0.001.

Brainstem sections were stained with haematoxylin and eosin as described in the methods, and vacuolation was quantified in the three nuclei. For WT mice treated with saline, the neuropil was normal in all cases, with no vacuoles present. In the facial nucleus (Figure 3 – E-H, R), a significant difference in the amount of vacuolation was evident between the treatment groups (Kruskal-Wallis p < 0.001). Substantial vacuolation was found in *SOD1*^*G93A*^ mice treated with both saline (0.179 [0.033] mm^2^) and free MP (0.168 [0.054] mm^2^), which were not significantly different from one another (p > 0.05). In contrast, vacuolation in *SOD1*^*G93A*^ mice treated with 2B3-201 (0.143 [0.041] mm^2^) was significantly lower than *SOD1*^*G93A*^ mice treated with saline (p < 0.05). The same pattern was found in the trigeminal (Figure 3 – A-D, Q) and hypoglossal nuclei (Figure 3 – I-L, S). However, in the trigeminal nucleus, in addition to being significantly lower than *SOD1*^*G93A*^ saline, vacuolation was significantly lower in *SOD1*^*G93A*^ mice treated with 2B3-201 compared with mice treated with free MP (p<0.05).

### Motor neuron counts

**Figure 4:**
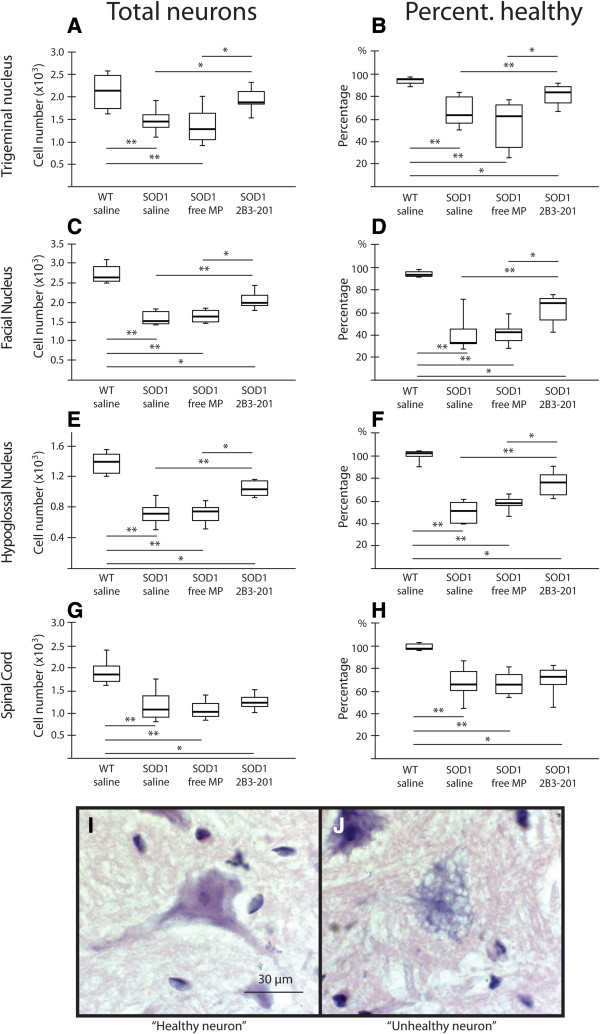
**Mutant** ***SOD1*** **-induced motor neuron loss is ameliorated by steroid treatment in cranial motor nuclei but not the lumbar spinal cord.** The total number of neurons **(A, C, E, G)**, and the percentage of morphologically normal neurons **(B, D, F, H)** are reduced in all *SOD1* ^*G93A*^ groups compared with WT mice. The *SOD1* ^*G93A*^ 2B3-201 treatment group showed a general improvement in total number of neurons and the proportion of those with a normal morphological appearance, compared with both saline and free MP in the brainstem nuclei. No improvement was seen in the spinal cord. Frames **I** and **J** show an example of a neuron defined as morphologically normal (healthy, **I**) and an abnormal neuron with significant vacuolation **(J)**. * p<0.01; ** p<0.001.

Motor neurons were counted using stereological techniques in the three motor nuclei to give the total number of motor neurons. The number of morphologically normal motor neurons (i.e. vacuole-free) was also counted to calculate the percentage of morphologically normal neurons. The number of neurons differed significantly between the treatment groups (Kruskal-Wallis p<0.001). In the facial nucleus (Figure 4 – C-D), WT mice had 2,720 [395] neurons in total, with 92.9 [3.6]% morphologically normal neurons. The total number of neurons was lower in all *SOD1*^*G93A*^ groups compared with WT mice: *SOD1*^*G93A*^ saline had 1,553 [317] neurons (p < 0.001), *SOD1*^*G93A*^ free MP 1,681 [294] neurons (p < 0.001), and *SOD1*^*G93A*^ 2B3-201 2,039 [277] neurons (p < 0.01), with more neurons in the *SOD1*^*G93A*^ 2B3-201 group than either the *SOD1*^*G93A*^ saline or free MP group (p < 0.01). Assessing number of healthy neurons as a percentage of the total number gives a more subtle assessment of disease status in the *SOD1*^*G93A*^ animals and drug treatment groups, and this measure also differed significantly between the treatment groups (Kruskal-Wallis p < 0.001). The percentage of morphologically normal neurons was much lower in all three *SOD1*^*G93A*^ groups compared with WT saline controls: 33.0 [12.7]% of neurons were morphologically normal in the *SOD1*^*G93A*^ saline group (p < 0.001), 42.3 [9.8]% in the *SOD1*^*G93A*^ free MP group (p < 0.001) and 67.9 [18.9]% in *SOD1*^*G93A*^ 2B3-201 mice (p < 0.01). *SOD1*^*G93A*^ 2B3-201 mice showed a significantly higher proportion of morphologically normal appearing neurons compared with *SOD1*^*G93A*^ saline (p < 0.001) and *SOD1*^*G93A*^ free MP mice (p<0.01). The same pattern was seen in the trigeminal (Figure 4 – A-B) and hypoglossal nuclei (Figure 4 – E-F).

Within a 760 μm block of the ventral horn of the spinal cord (Figure 4 – G-H), WT mice had a total of 1,856 [342] neurons, with 93.5 [4.2]% morphologically normal neurons. A significant difference in the number of neurons was found between treatment groups (Kruskal-Wallis p < 0.001). All *SOD1*^*G93A*^ treatment groups had fewer total neurons compared with WT control mice: 1,083 [469] neurons in the *SOD1*^*G93A*^ saline group (p < 0.001), 1,032 [282] in the *SOD1*^*G93A*^ free MP group (p < 0.001), and 1,229 [200] in the *SOD1*^*G93A*^ 2B3-201 group (p < 0.01). A significant difference was also evident between treatment groups in terms of morphologically healthy neurons (Kruskal-Wallis p < 0.001). A smaller percentage of morphologically normal neurons was observed in the three *SOD1*^*G93A*^ treatment groups compared with WT mice: 60.8 [15.5]% in *SOD1*^*G93A*^ saline mice (p < 0.001), 60.9 [15.2]% in *SOD1*^*G93A*^ free MP mice (p < 0.001), and 67.0 [11.1]% in *SOD1*^*G93A*^ 2B3-201 mice (p < 0.01). However, unlike the three cranial motor nuclei above, no difference was found between any of the *SOD1*^*G93A*^ treatment groups, either for total neurons, or percentage morphologically normal neurons (p>0.05).

### Glial cell contribution

Glial cells are know to contribute to neuronal pathology in both humans, and animal models of ALS [10, 11], so we postulated that 2B3-201 treatment exerted its effects by altering the inflammatory environment of the CNS, through astrocytes or microglia.

Sections of the cranial motor nuclei and spinal cord were stained using an antibody against Iba1 for activated microglia, and the intensity of stain was quantified as described in the methods. Iba1 is expressed in all microglia, irrespective of activation state. However, staining intensity significantly upregulated on activation in response to injury or cell death. We processed the tissue such that low levels of basal expression in WT animals were below the detection threshold of this technique (as demonstrated by the very low staining intensity in WT tissue). Thus the microglia that were counted with our staining method were mostly of the “activated” variety. The numbers of Iba1-positive microglia were counted in the grey matter of the cranial motor nuclei and ventral horn of the spinal cord.

**Figure 5:**
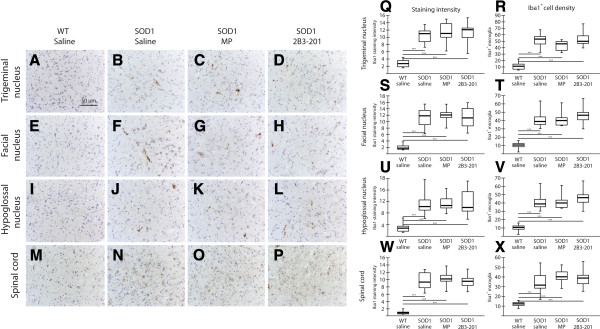
**Iba1 positive microglia are not altered following steroid treatment.** WT Iba1 immunoreactivity was very low in the cranial motor nuclei **(A, E, I)**, and in the spinal cord **(M)**. *SOD1* ^*G93A*^ mice treated with saline **(B, F, J, N)**, free MP **(C, G, K, O)** and 2B3-201 **(D, H, L, P)** showed much stronger staining for Iba1, both in the three brainstem nuclei and the spinal cord. No difference was found in immunoreactivity between *SOD1* ^*G93A*^ treatment groups. Panels **Q**, **S**, **U** and **W** show quantification of Iba1 staining intensity in the brainstem motor nuclei and spinal cord, and panels **R**, **T**, **V** and **X** show the density of Iba1^+^ microglia in these same regions, showing increased expression in all SOD1^G93A^ treatment groups compared with WT mice, but no group differences among treatments. Scale bar applies to all images. * p<0.05; ** p<0.01; *** p<0.001.

In the facial nucleus (Figure 5 – E-H, S, T), WT Iba1 staining intensity was 1.8 [0.1] units, and on average 10.6 [3.4] Iba1-positive microglia were evident per ROI. A significant difference in Iba1 staining intensity was found between the treatment groups (Kruskal-Wallis p < 0.001). Iba1 staining intensity was greater for all *SOD1*^*G93A*^ mice compared with WT, with an average of 12 units (p < 0.001) and significantly more Iba1-positive microglia, with an average of 41 microglia per ROI, were seen in the three *SOD1*^*G93A*^ groups compared with WT mice (p < 0.001). However, there were no differences in Iba1 staining intensity or number of microglia between *SOD1*^*G93A*^ treatment groups (p > 0.05). The same pattern was seen in the trigeminal nucleus (Figure 5 – A-D, Q, R), the hypoglossal nucleus (Figure 5 – I-L, U, V) and the ventral horn of the spinal cord (Figure 5 – M-P, W, X).

Serial brainstem and spinal cord sections were stained using an antibody against GFAP to reveal astrocytes, and the intensity of stain was quantified as described for Iba1 above. It is important to note that GFAP is only expressed in a subset of astrocytes, and its expression is increased by inflammatory signaling. Unlike the white matter, where there is a large population of GFAP-positive astrocytes, few are typically seen in the grey matter of WT mice (as demonstrated by the low staining intensity in WT tissue). The number of GFAP positive astrocytes was counted in the grey matter of the cranial motor nuclei and ventral horn of the spinal cord.

**Figure 6:**
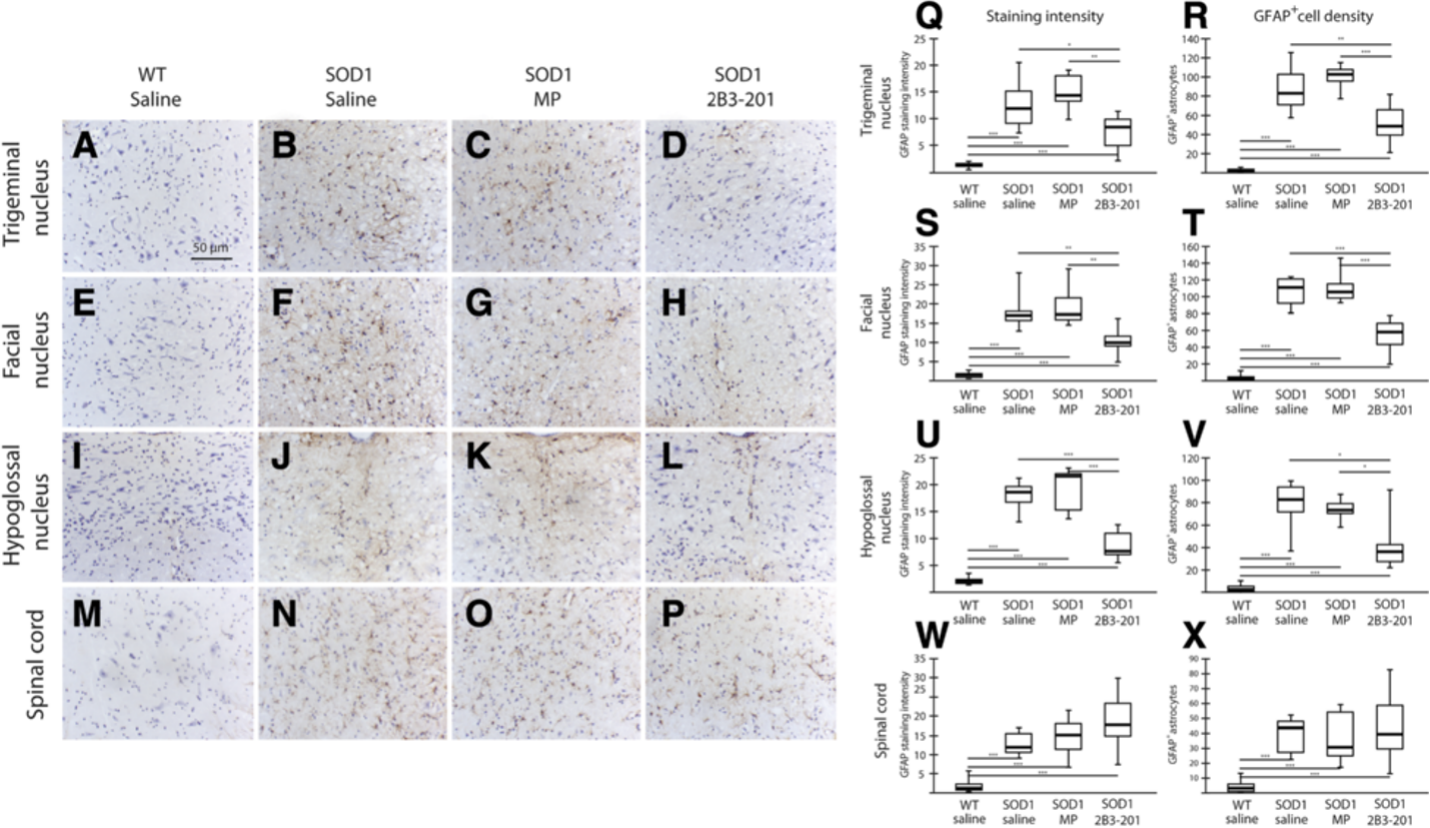
**GFAP positive astrocytes reduced by steroid treatment.** There is very little GFAP staining in the grey matter in WT mice, either in the cranial motor nuclei **(A, E, I)**, or spinal cord **(M)**. In the *SOD1* ^*G93A*^ saline group there was a marked GFAP up-regulation in the trigeminal **(B)**, facial **(F)** and hypoglossal **(J)** nuclei, as well as the ventral spinal cord **(N)**. A similar staining pattern is observed in *SOD1* ^*G93A*^ mice treated with free MP **(C, G, K, O)**. GFAP staining in *SOD1* ^*G93A*^ 2B3-201 mice, however, was significantly reduced in all cranial motor nuclei **(D, H, L)**, but not in the ventral spinal cord **(P)**. Panels **Q**, **S**, **U** and **W** show quantification of GFAP staining intensity in the brainstem motor nuclei and spinal cord, and panels **R**, **T**, **V** and **X** show the density of GFAP^+^ astrocytes in these same regions. There is increased expression in all SOD1^G93A^ treatment groups compared with WT mice, but SOD1^G93A^ mice treated with 2B3-201 have less of a GFAP response compared with those treated with saline and free MP. Scale bar applies to all images. * p<0.05; ** p<0.01; *** p<0.001.

In the facial nucleus (Figure 6 – E-H, S, T), WT saline GFAP staining intensity was 1.5 [0.9] units, consisting of 2.6 [2.6] GFAP-positive astrocytes per ROI. A significant difference in the GFAP staining intensity was found between treatment groups (Kruskal-Wallis p < 0.001). The GFAP staining intensity was much higher in all *SOD1*^*G93A*^ treatment groups compared with WT mice (p < 0.001). In addition GFAP signal intensity was significantly lower in *SOD1*^*G93A*^ 2B3-201 (10.0 [2.3]) compared with both *SOD1*^*G93A*^ saline (17.0 [2.4]) (p < 0.01) and *SOD1*^*G93A*^ free MP (17.3 [5.7]) (p < 0.01). The number of reactive astrocytes was also significantly different between groups in the facial nucleus (Kruskal-Wallis p < 0.001). The number of GFAP^+^ astrocytes was greater in the three *SOD1*^*G93A*^ treatment groups compared with WT mice (p < 0.001). *SOD1*^*G93A*^ 2B3-201 mice (58.1 [25.3]) had fewer astrocytes compared with both *SOD1*^*G93A*^ saline (111 [28.6]) (p < 0.001) and *SOD1*^*G93A*^ free MP mice (105.6 [17.3]) (p < 0.001). The same pattern was seen between the treatment groups in the trigeminal (Figure 6 – A-D, Q, R) and hypoglossal nuclei (Figure 6 – I-L, U, V). In the ventral horn of the spinal cord (Figure 6 – M-P, W, X), WT GFAP staining intensity was 1.1 [1.3] units, and 3.1 [4.8] reactive astrocytes were detected per ROI. A significant difference in GFAP staining intensity and the number of GFAP^+^ astrocytes overall was also evident between the groups (Kruskal-Wallis p < 0.001): significantly higher GFAP immunoreactivity was seen in all three *SOD1*^*G93A*^ groups compared with WT (p < 0.001), an average of 15 units or 38 cells/ROI between the three *SOD1*^*G93A*^ treatment groups. However, unlike for the cranial motor nuclei, no significant differences were found between *SOD1*^*G93A*^ treatment groups with regards to either GFAP staining intensity or number of astrocytes.

## Discussion

Overall, we present data showing that glutathione PEGylated liposomal methylprednisolone (2B3-201) reduced brainstem neuronal loss and degeneration, and attenuated brainstem astrocyte reactivity in the *SOD1*^*G93A*^ mouse model of ALS.

There was no significant difference between *SOD1*^*G93A*^ mice treated with 2B3-201 compared with saline in regards to our behavioural outcomes (weight and rotarod). However, Rotarod score does not reflect all of the various motor neuron populations relevant for ALS (i.e. spinal, cranial and cortical); rotarod performance requires balance and motor skills of the fore- and hind-limbs, and, as a consequence, it will primarily reflect physiology of lumbar motor neurons (for hind limbs), cervical motor neurons (for fore limbs) and cerebellar neurons (for balance). Therefore, a significant improvement in neuronal health and alterations in the inflammatory environment in the cranial nuclei would not be reflected in the rotarod score. As ALS progresses there is substantial involvement of cranial neuropathy to clinical symptoms, many of which ultimately contribute to early death of patients. The function of these motor nuclei is not easy to determine with standard behavioral testing, for example studies of ability to eat (a test of trigeminal, facial, glossopharyngeal, vagus and hypoglossal nerve function) are confounded by affect, appetite or general mobility, which may be separately altered in neurodegenerative disease. MRI affords an opportunity to examine the structural integrity of the higher motor centres.

As has been shown in previous studies from our group and elsewhere [3–5], *SOD1*^*G93A*^ mice had T_2_ hyperintensity in the brainstem motor nuclei, which was significantly attenuated by 2B3-201 treatment, suggesting that 2B3-201, but not free steroid, ameliorates brainstem pathology. The increased *T*_2_ signal intensity has been shown to correlate with vacuolization associated with neuronal cell death, as well as astrocyte and microglia activation [3, 4]. To discover whether the positive effects of 2B3-201 on T_2_ MRI signal correlated with increased neuronal cell survival we examined post-mortem tissue with histology and immunohistochemistry.

We showed a specific therapeutic effect for 2B3-201 (but not free MP) in reducing not only brainstem vacuolation, but also reducing the neuronal loss in these nuclei, and increasing the number of histologically “healthy” appearing neurons on histology, thus confirming the T_2_ MRI findings. However, there was no therapeutic effect, either for free or packaged steroid, for spinal (ventral horn) neuronal loss. There are no published reports to date of glucocorticoid treatment in *SOD1* mouse models of ALS, which is surprising given the interest many groups have in the influence of neuroinflammation in the disease. The results presented here are consistent with reports in the Wobbler mouse, showing that corticosterone and 21-aminosteroids can have therapeutic effect in models of motor neuron degeneration [17, 22, 23]. However, the pathology in the Wobbler mouse is more diffuse and is now regarded as less relevant to ALS.

Glucocorticoids tend to up-regulate anti-inflammatory gene expression [24] and interfere with regulation of pro-inflammatory genes [25]. To discover whether the effects of the 2B3-201 administration could be attributed, at least in part, to the suppression of the CNS inflammatory response we examined the morphology of astrocytes and microglia. Glial cells are know to contribute to neuronal pathology in both humans, and animal models of ALS [10, 11], so we postulated that 2B3-201 treatment exerted its effects by altering the inflammatory environment of the CNS, through astrocytes or microglia. In line with previous reports, we found that there was marked activation of astrocytes and microglia in both the cranial motor nuclei and ventral horn of the spinal cord in *SOD1*^*G93A*^ mice treated with saline [10, 11]. There was no effect of free MP administration on microglial activation at any site, but administration of 2B3-201 to *SOD1*^*G93A*^ mice significantly reduced GFAP immunoreactivity in the cranial nuclei, but not in the ventral horn of the spinal cord. Given the importance of microglia in the initiation of neurodegeneration in SOD1 mice, it is surprising that preservation of neurons was not associated with obvious changes in microglia reactivity. However, much of the work done to date in *SOD1*-ALS (and indeed ALS in general) in regard to neuron-microglia interactions has been correlational, and our understanding of the relationship between microglial activation and neuronal degeneration in this model is still limited. The dissociation between microglial activation and neuronal survival, however, is strong evidence that factors other than microglia are relevant for neuronal survival in *SOD1*^*G93A*^ mice.

The astrocyte-specific anti-inflammatory effect of 2B3-201 raises the possibility that the positive effects of the drug may be mediated by improving astrocyte function. Support for this notion comes from recent work showing astrocyte dysfunction in the *SOD1*^*G93A*^ mouse alters motor neuron function at a gene transcription level [26]. Studies from other neuroinflammatory models, such as experimental autoimmune encephalomyelitis (EAE), have indicated that reactive, GFAP-positive astrocytes can have impaired glutamate metabolism, with decreased action of glutamate dehydrogenase and glutamine synthetase, which results in higher glutamate concentration and potential excitotoxicity [27]. It would be of interest, therefore, to investigate whether the effect on neuronal survival and reduced vacuolation shown here is mediated by metabolic changes induced by a phenotypic shift of astrocytes.

The superior efficacy of 2B3-101, glutathione PEGylated liposomal methylprednisolone, compared to the free methylprednisolone highlights the importance of CNS targeting of therapeutic agents. A number of studies have shown altered BBB permeability as a part of disease pathogenesis in *SOD1* rodent models [28–30], but such dysfunction appears to occur at the end of the disease, if at all, and is unlikely to facilitate drug entry to the brain. In fact, recent work indicates that the function of the p-glycoprotein efflux transporter at the BBB is upregulated as part of disease progression in the *SOD1* mice [31], which would greatly reduce the therapeutic potential for a non-encapsulated steroid treatment. This may go some way to explain why free prednisolone was not effective as a treatment in ALS patients [18].

When interpreting these data, it is important to note that the assessment of neuronal health in this report is a purely morphological one. Whilst highly vacuolated cells are highly likely to have functional deficits, this could be confirmed with electrophysiological analysis. Also, the analysis of phenotypic changes in astrocytes and microglia used here is only based on one, well-validated, marker each using immunohistochemistry. One important goal for the future will be to use other markers to delve deeper in the phenotype of these cell types and their associated functional state.

Overall, the therapeutic effect of 2B3-201 was limited to the cranial motor areas – substantial motor neuron loss was evident in all three *SOD1*^*G93A*^ groups within the ventral horn of the spinal cord, and no amelioration was found with 2B3-201 treatment for either neuronal, tissue integrity (vacuolation) or inflammatory parameters. This was surprising, as previous work using glutathione PEGylated liposomes encapsulating a fluorescent tracer has found a high delivery to the spinal cord, in addition to higher cerebral structures [32]. It is unclear from this work why 2B3-201 did not affect the spinal cord in this model. However, It is worth noting that while most studies investigating potential therapeutic compounds for ALS are focused towards ameliorating the spinal cord pathology, death in ALS is typically due to failure of the respiratory muscles, which may have a brainstem component [33, 34]. Moreover, towards the end of disease (or at earlier disease points for patients with bulbar-onset ALS), dysphagia may occur due to bulbar degeneration, potentially causing malnutrition unless the patients undergo gastrostomy feeding [35]. Malnutrition may have consequences to other body systems, and associated dysphagia may lead to aspiration pneumonia [36]. Dysphagia has also been highlighted as a major factor in impairing quality of life for ALS patients [37]. Therefore, targeting brainstem pathology in ALS patients has important therapeutic potential. We chose to employ a treatment regime beginning at 60 days, and it remains possible that earlier intervention with the steroid may have had more of a beneficial effects on the spinal pathology should it have been given earlier. It remains unclear at what point cell death becomes irreversible in *SOD1*^*G93A*^ mouse pathology.

## Conclusions

This work has shown that a CNS-targeted anti-inflammatory steroid treatment (glutathione PEGylated liposomal methylprednisolone) can significantly ameliorate pathology in a *SOD1*^*G93A*^ mouse model of ALS, reducing signs of neurological damage by as much as 40%. It also confirms the potential of MRI as a biomarker of disease progression and drug efficacy in ALS [38]. History suggests that caution should be exercised in assuming translation of benefit from the *SOD1* mouse to human ALS [39]. Nonetheless, we highlight an important principle, namely that drugs with little or no effect following standard systemic administration may have significant effects on the pathophysiology when targeted to the CNS through optimized delivery.

## Materials and methods

### Animals and treatments

Female *SOD1*^*G93A*^ mice and wild-type (WT) C57BL/6 littermates were sourced from the in-house breeding colony at the University of Oxford. Male *SOD1*^*G93A*^ mice were used to maintain the breeding colony, and were not used in experiments. Animals were genotyped at 40 days of age, using a DirectPCR Lysis Reagent (Viagen, York, UK) following standard manufacturer’s directions to extract DNA and standard PCR was performed using primers for human *SOD1*^*G93A*^ and *IL-6* (positive control), as described on the Jackson laboratory website (http://jaxmice.jax.org/strain/002726.html). Animals were then assigned to one of four groups: (1) WT saline (n = 10); (2) *SOD1*^*G93A*^ saline (n = 8); (3) *SOD1*^*G93A*^ free methylprednisolone (MP) (n = 9); and (4) *SOD1*^*G93A*^ 2B3-201 (n = 10). 2B3-201, glutathione PEGylated liposomal MP, was prepared as previously described [21]. Treatments were started on day 60 and animals were treated with either saline (groups 1, 2), 10 mg/kg free MP (group 3) or 10 mg/kg MP packaged in 2B3-201 (group 4) by intravenous injection once a week for 9 weeks (until day 116). All animal procedures were conducted with UK Home Office approval (licence number 30/2524).

### Behavioural testing

Immediately prior to each injection, animal weights were recorded and behaviour was tested on a rotarod using an accelerating paradigm, starting at 4 rotations/minute and accelerating to 40 rotations/minute over 5 minutes. The time taken for an animal to fall from the apparatus was recorded in triplicate for each animal, and the longest time was used in the analysis.

### Magnetic resonance imaging (MRI)

For MRI, all animals were anaesthetised using 2.5% isofluorane (IsoFlo®, Abbot Laboratories, Maidenhead) for induction, and subsequently maintained with 1-2% isoflurane in 30% O_2_ and 70% NO_2_, a flow rate of 1.5 L/min. MRI data were acquired on a 7T magnet with a Varian Inova spectrometer (Varian Inc., subsidiary of Agilent Technologies, Santa Clara, CA, USA). *T*_2_-weighted images were acquired using a fast spin-echo multislice (fSEMS), sequence: TR = 3000 ms; TE = 61.9 ms; FOV = 25 x 25 mm; Matrix size = 128 x 128; number of slices = 11; averages = 2; slice thickness = 1 mm; scanning time = 3 mins 12 sec. ROIs were placed over the three motor nuclei (trigeminal, facial and hypoglossal), as well as a control region on the same slices as the nuclei (within the brainstem, but outside of the nuclei of interest), and intensities were measured as (ROI-control)/control.

### Tissue preparation

Following MRI, animals were terminally anaesthetized and perfusion-fixed with PLP_light_ (75mM lysine HCL, 10 mM di-sodium hydrogen orthophosphate, 10 mM sodium periodate, 2% PFA, 0.05% gluteraldehyde), and, after removal of the brain and spinal cord, the tissue was further fixed by immersion in the same fixative. Tissue was cryo-protected with 30% sucrose, and then frozen in OCT (Sigma) and 10-μm-thick sections were cut; each adjacent section was 150μm apart. Immunohistochemistry was performed using a standard protocol for frozen sections using the following primary antibodies: rabbit polyclonal GFAP (1:1,000, 24°C, 4 hours) and goat polyclonal Iba-1 (1:500, 24°C, 3 hours). All primary antibodies were diluted in PBS with 0.1% Tween, all washes were with PBS. Secondary antibodies, either biotinylated horse anti-goat IgG or goat anti-rabbit IgG, were diluted 1:100, and incubated on the tissue sections for 1 hour at 24°C. Biotin was bound to HRP-Streptavidin (ABC kit, Vectorstain), and applied to the tissue for 1 hour at 24°C, and reaction product developed with DAB (Sigma). Tissue processing was optimised so that very little GFAP or Iba1 staining in brainstem nuclei and spinal cords in wild type animals, but good staining in inflammatory tissue. Tissue was also stained with haematoxylin and eosin (H&E).

### Vacuolation/immunohistochemistry analysis

Vacuolation was assessed in H&E sections. Photographs were taken of left and right brainstem or spinal cord hemispheres from 2 sections per brain at 20x magnification. For selection of sections for analysis, the largest two cross sections through the nucleus were selected for each animal. For assessment of vacuolation, images were binarised by thresholding using ImageJ, creating a black mask over the tissue (value 0) and a bright mask over the vacuoles (value 266). Amount of tissue vacuolation was then calculated as follows: the area of tissue where vacuolation was present was measured (mm^2^), and within this the binarized mask was averaged on a pixel-by-pixel basis to obtain a measure of the proportion of this area affected by vacuolation. By dividing the area by the proportion affected, the total area of tissue replaced by vacuoles was calculated. Vacuolation of WT mice was measured in the same way as the *SOD1*^*G93A*^ tissue as a baseline control, except that an arbitrary ROI was used, placed around the boundary of the nucleus, with the size consistent with the ROI for *SOD1*^*G93A*^. We have previously published using this method, showing excellent group separation between *SOD1*^*G93A*^ and wild type control throughout much of the extent of the disease [5].

For quantification of both GFAP and Iba1 immunostaining, the two largest cross sections of the brainstem nuclei in both hemispheres (total 4 regions), or the left and right ventral horns (total 4 regions) were photographed at 20x magnification, using Leica Firecam software. In ImageJ, the image was deconvolved to split the brown DAB channel from the blue haematoxylin channel, and then the average staining intensity was measured in an ROI of diameter 500 units on ImageJ placed over the motor nucleus. In addition to the average staining intensity, the average number of astrocytes stained with GFAP and microglia stained with Iba1 were counted in the same regions of the brainstem and spinal cord. All histological analysis, including selection of sections for analysis, was performed blinded to treatment group.

### Neuronal number estimation

The number of neurons was counted in the trigeminal, facial and hypoglossal nuclei, and a 760 μm segment of the lumbar spinal cord, using an optical fractionator method after West et al. [40]. Brainstem and spinal cord sections were cut at 10 μm (post-processing thickness 6 μm), adjacent sections were 150 μm apart, and stained with H&E. Cells were randomly sampled using a sampling grid of squares 75 μm x 75 μm in dimension, separated by 150 μm in x and y directions, using *StereoInvestigator* software using a x20 objective lens. Cells were only counted when nuclei appeared within the thickness of the section. The total number of neurons was counted, and the number of morphologically normal neurons was also recorded; these quantities were used to calculate the percentage of healthy neurons. Morphologically normal neurons display a regular staining pattern with H&E, whereas abnormal neurons show intracellular vacuolation and often have a withered, pyknotic appearance (see Figure 4).

### Statistical analysis

For the behavioural data, repeated measure ANOVAs (within-subjects factor time, between subjects factor treatment) and Holm-Bonferroni t-tests were carried out at each time point. Greenhouse-Geisser correction was used because Mauchly’s test of sphericity was significant (p<0.001) Area under the curve (AUC) analysis was also performed using the trapezoid rule, and group means were compared using a one-way ANOVA followed by Bonferroni-corrected t tests. MRI and histology data were not normally distributed, and could not be normalized by transformation; therefore Kruskal-Wallis analysis was used to investigate differences between treatment groups in the three brainstem motor nuclei. Individual comparisons were made using Mann-Whitney U tests (corrected for multiple comparisons). SPSS (SPSS Inc., Chicago, USA) was used for all parametric and non-parametric statistical analysis. Data are presented as mean ± standard error for behavioural data and median [interquartile range] for all other data. Similarly means and standard errors are graphically represented by bar graphs with standard error bars, whereas box and whisker plots are used and display the median (strong central band), interquartile range (box) and range (whiskers) for all corresponding non-parametric analyses.

## Abbreviations

ALS: Amyotrophic lateral sclerosis
CNS: Central nervous system
GFAP: Glial fibrillary acidic protein
MN: Motor neuron
MP: Methylprednisolone
MRI: Magnetic resonance imaging
PEG: Polyethylene glycol
ROI: Regions of interest
SOD: Superoxide dismutase
WT: Wild type.
